# Associations Between Delayed Cerebral Ischemia in Spontaneous Subarachnoid Hemorrhage and Dysfunction of Autonomic Cardiovascular Modulation Compared to Transcranial Doppler Ultrasound Findings

**DOI:** 10.3390/diagnostics16132125

**Published:** 2026-07-07

**Authors:** Matthias C. Borutta, Chiara Vetter, Florian Kraemer, Stefan T. Gerner, Kosmas Macha, Ludwig Singer, Tobias Engelhorn, Arnd Doerfler, Stefan Schwab, Julia Koehn

**Affiliations:** 1 Department of Neurology, Friedrich-Alexander-University of Erlangen-Nuremberg (FAU), Schwabachanlage 6, 91054 Erlangen, Germany; chiara.vetter@uk-erlangen.de (C.V.); florian.kraemer@uk-erlangen.de (F.K.); stefan.gerner@uk-erlangen.de (S.T.G.); kosmas.macha@uk-erlangen.de (K.M.); stefan.schwab@uk-erlangen.de (S.S.); 2 Department of Neuroradiology, Friedrich-Alexander-University of Erlangen-Nuremberg (FAU), Schwabachanlage 6, 91054 Erlangen, Germany; ludwig.singer@uk-erlangen.de (L.S.); tobias.engelhorn@uk-erlangen.de (T.E.); arnd.doerfler@uk-erlangen.de (A.D.); 3 Department of Neurology, ANregiomed, Escherichstraße 1, 91522 Ansbach, Germany

**Keywords:** spontaneous subarachnoid hemorrhage, delayed cerebral ischemia, autonomic nervous system, heart rate variability, autonomic dysregulation, transcranial Doppler ultrasound, vasospasm

## Abstract

**Background**: This study aims to assess associations between occurrence of delayed cerebral ischemia (DCI) in spontaneous subarachnoid hemorrhage (SAH) and possible dysfunction of autonomic cardiovascular modulation compared to transcranial Doppler ultrasound (TCD). **Methods**: In this prospective observational study, 53 patients with spontaneous SAH were enrolled, and 17 patients met DCI criteria, i.e., new cerebral infarction > 72 h after SAH onset on follow-up CT scans. Autonomic modulation as well as TCD-frequencies were monitored within 24 h after SAH onset and then daily until day 10. From 5 min time-series of R–R-interval (RRI) and blood-pressure (BP) recordings, parameters of sympathetic, parasympathetic and total autonomic cardiovascular modulation were calculated, including time- and frequency-domain parameters. Data were compared between patients with and without DCI. Further subgroup analyses were performed according to functional outcome after 3 to 6 months (i.e., favorable outcome, modified Rankin Scale (mRS) ≤ 3 vs. unfavorable outcome, mRS > 3) regardless of DCI. **Results**: RRI and BP values as well as TCD frequencies did not differ between patients with and without DCI. Compared to No DCI patients, the cohort of DCI patients had significantly lower values of sympathetic modulation (RRI-LF powers, SBP-LF powers) on days 5 and 9 after SAH, significantly lower values of total autonomic modulation (RRI-SD, RRI-CV, RRI-total powers) and insignificantly lower values of parasympathetic modulation (RMSSDs, RRI-HF powers) on day 5 after SAH. Parameters of sympathetic, parasympathetic, and total autonomic modulation did not differ significantly between patients with favorable and unfavorable outcomes, but showed slightly lower values in the unfavorable outcome group. Yet, additionally calculated value of normalized RRI-LF and normalized RRI-HF powers, as well as LF/HF ratios were significantly different in the unfavorable outcome cohort. **Conclusions**: Not only within the acute phase, but also during the first days after disease onset, spontaneous SAH induces a decrease in sympathetic, parasympathetic and total autonomic cardiovascular modulation. In contrast to standard diagnostic evaluation for detecting clinically relevant vasospasms—i.e., TCD—autonomic dysfunction was associated with development of DCI and poor clinical outcome. Thus, assessment of heart rate variability may predict augmented risk of cardiovascular complications and may represent a promising adjunctive marker within multimodal neuromonitoring in SAH patients.

## 1. Introduction

The incidence of spontaneous subarachnoid hemorrhage (SAH), a subtype of intracranial bleeding, has decreased over the past decades in industrialized nations—ranging from 14% (USA) to 59% (Japan) since the 1950s [1,2]. Nevertheless, despite substantial developments and improvements in the acute management of cerebral aneurysms, mortality has remained at unchanged relevant levels [1,2]. And although treatment of early brain injury (EBI), i.e., securing the bleeding source, managing obstructive hydrocephalus, preventing cerebral herniation, and dealing with early-onset cardiorespiratory complications, evolved, a substantial portion of patients still die before hospital admission or during the subsequent, early course of treatment [1,2,3].

This fact may partly be attributed to complications of secondary brain injury (SBI), among which cerebral macro- and microvasospasms and associated delayed cerebral ischemia (DCI) are decisive determinants of morbidity, mortality, and functional outcome [1,2].

Standard diagnostic evaluation for detecting clinically relevant vasospasms relies on daily bedside transcranial Doppler ultrasound (TCD). However, TCD results require cautious interpretation: despite high sensitivity (approx. 90%), the technique has only moderate specificity (approx. 70%), detects only proximal macrovasospasms, and constrained temporal bone windows may render sonographic monitoring impossible [2,4]. Furthermore, while vasospasms occur frequently, only about 30% result in DCI, as its etiology is multifactorial and depends, among other factors, on the amount and distribution of subarachnoid blood as well as autonomic, neuroendocrine, and inflammatory mechanisms [1,4]. To better assess early microcirculatory dysfunction, computed tomography (CT) perfusion imaging may be used; however, its indication must also be critically evaluated due to the risks associated with contrast administration (especially in patients with renal impairment), radiation exposure, and the significantly increased complications during transport of critically ill patients. As a result, routine perfusion imaging is not feasible for general vasospasm monitoring [4,5]. Additionally, recent research suggests that DCI can occur even in the absence of detectable vasospasm on TCD, CT angiography, or even digital subtraction angiography [6].

Other evolving diagnostic techniques comprise expanded multimodal neuromonitoring through invasive techniques—e.g., parenchymal probes for PtbO_2_ measurement and microdialysis, or noninvasive methods such as quantitative electroencephalography or near-infrared spectroscopy. However, these modalities are not sufficiently validated for routine clinical use, and therefore (so far) not recommended in various national and international guidelines [4].

Consequently, there is urgent need for a diagnostic tool suitable for early detection of cerebral vasospasm and DCI that meets the following criteria: noninvasive, ideally continuous, bedside applicable monitoring, preferably without requiring additional hardware. Heart rate variability (HRV) monitoring meets these constraining requirements [7].

Given the complex pathophysiology of spontaneous SAH—with cerebral and extracerebral complications related to EBI and SBI—analysis of the autonomic nervous system offers a unifying perspective on the possibly severely disrupted autoregulation and homeostasis observed throughout the disease course [1,7].

Previous studies have demonstrated that autonomic dysfunction may develop following SAH, and that the clinical severity of SAH (measured by the Hunt and Hess scale) correlates with autonomic impairment within the first 24 h after onset as well as with functional outcomes at discharge [8,9].

The present study therefore aims (i) to investigate whether HRV assessment may predict DCI compared to TCD, and (ii) whether possible abnormalities of autonomic cardiovascular modulation during the disease course are associated with functional outcomes at three to six months after SAH.

## 2. Materials and Methods

### 2.1. Patient Selection

**Figure 1:**
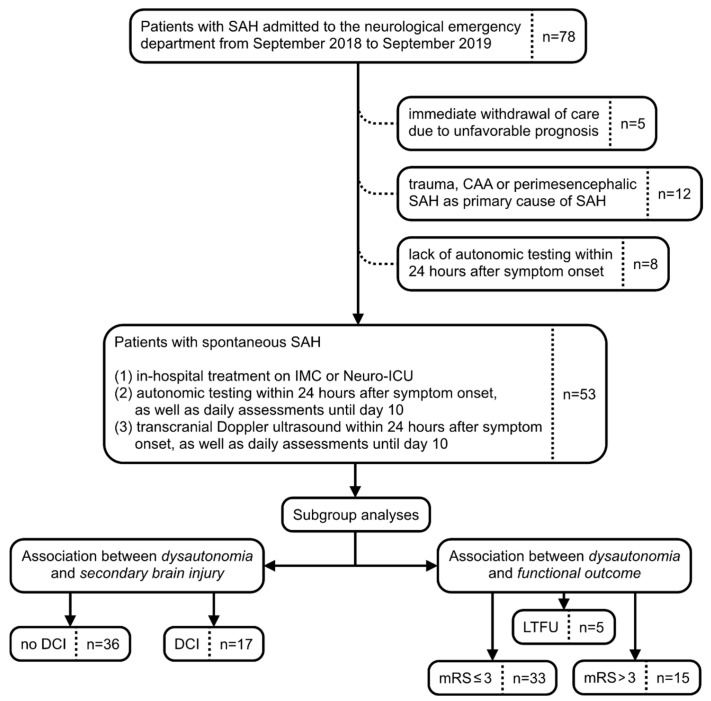
Flowchart of study participants and subgroups with regard to secondary brain injury and functional outcome. [*SAH*, subarachnoid hemorrhage; *CAA*, cerebral amyloid angiopathy; *IMC*, intermediate care unit; *ICU*, intensive care unit; *DCI*, delayed cerebral ischemia; *mRS*, modified Rankin Scale; *LTFU*, lost to follow-up].

Between 09/2018 and 09/2019 all SAH patients admitted to the department of Neurology of the Friedrich-Alexander University Hospital of Erlangen (FAU), Germany, were screened for eligibility. Patients were excluded from study participation in cases of (1) immediate withdrawal of care due to unfavorable prognosis, (2) trauma or clear cerebral amyloid angiopathy as the primary SAH cause, and (3) lack of autonomic testing within 24 h after symptom onset. Inclusion criteria consisted of (1) in-hospital treatment in the intermediate care unit, i.e., stroke unit, or neurointensive care unit, (2) autonomic testing within 24 h after symptom onset and daily assessments until day 10, and (3) TCD within 24 h after symptom onset, as well as daily assessments until day 10. Patient screening, including inclusion and exclusion criteria, are shown in Figure 1.

The study protocol was approved by the institutional review board of the faculty of medicine of the University of Erlangen–Nuremberg. Before patients were enrolled, written informed consent had been obtained from all participants or their legal representatives according to the Declaration of Helsinki.

### 2.2. Clinical Parameters

SAH diagnosis was made by the treating physicians upon cranial computed tomography (CT) imaging (SOMATOM Definition AS+, Siemens, Erlangen, Germany). Furthermore, indication of digital subtraction angiography for detection of the source of bleeding potentially followed by endovascular or surgical aneurysm treatment if applicable was made by the treating physicians. Patient management was carried out in accordance with institutional treatment protocols, and in line with the most current clinical guidelines for SAH management at the time of treatment.

An independent neuroradiologist who was blinded to clinical parameters and study process reviewed CT scans. Documented radiological parameters, e.g., concomitant intracerebral hemorrhage (ICH), intraventricular hemorrhage (IVH), and acute hydrocephalus have been reported in an earlier study on the same patient cohort [9].

Definite diagnosis of DCI was made by the independent neuroradiologist during hospital stay. For this study, the diagnosis of DCI was not made on the basis of clinical parameters (e.g., persistent clinical deterioration with a new focal neurological deficit), but solely on corresponding criteria on follow-up CT scans, i.e., new cerebral infarction more than 72 h after SAH onset, not explained by other causes [4].

Data on demographic parameters, prior comorbidities and premedication were retrieved from the institutional electronic databases. Assessed clinical parameters that were used for further analyses consisted of (1) pre-SAH modified Rankin Scale (pre-mRS, ranging from 0, i.e., functional independence, to 5, i.e., severe disability requiring constant nursing care) to grade patient’s disability prior to hospital admission, (2) Hunt and Hess score (clinical score, ranging from 1, i.e., mild headache, to 5, i.e., coma or decerebrate posturing), (3) modified Fisher scale score (radiological score for classifying the amount of subarachnoid hemorrhage, ranging from 0, i.e., no hemorrhage, to 4, i.e., thick subarachnoid and intraventricular hemorrhage) to evaluate clinical condition upon hospital admission, as well as (4) the modified Rankin Scale (ranging from 0 to 6, with a dichotomization of favorable outcome, i.e., mRS ≤ 3, and unfavorable outcome, i.e., mRS > 3) three to six months after hospital discharge to assess functional outcome (Table 1 and Table 2).

### 2.3. Transcranial Doppler Ultrasound

Bedsided TCD assessments were obtained daily until day 10 as a noninvasive method for indirect measurement of the presence of cerebral vasospasms as a recommended, standard of care diagnostic tool [4]. Two independent neurologists with sufficient experience who were blinded to the study process carried out TCD examinations. Testing was conducted using a pulsed-wave Doppler sonography instrument with a 2.0 MHz transducer and an energy output level corresponding to 100 mW per squared spatial peak-temporal average intensity (Dolphin/4d, VIASONIX, Ra’Anana, Israel). Reported frequencies were obtained from the proximal middle cerebral artery (depth of 40 to 65 mm), anterior cerebral artery (depth of 70 to 75 mm), and posterior cerebral artery (depth of 55 to 70 mm) with a sample volume size of 8 to 10 mm in the axial and 5 mm in the lateral direction, as well as the basilar artery (transforaminal in a depth of 90 to 120 mm) [10,11]. Frequencies were graded using the following criteria: <3 kHz (i.e., about <120 cm/s) indicating no vasospasm, ≥3 kHz (i.e., about ≥120 cm/s) borderline vasospasm, ≥4 kHz (i.e., about ≥160 cm/s) significant vasospasm, and ≥6 kHz (i.e., about ≥240 cm/s) critical vasospasm [12].

### 2.4. Autonomic Cardiovascular Modulation

Parameters of cardiovascular autonomic modulation were obtained within 24 h after SAH onset and then consecutively on a daily base until day 10. Measurements were performed bedsided between 9:00 a.m. and 2:00 p.m. to ensure comparable conditions. We noninvasively monitored R–R intervals (RRIs) via 3-lead electrocardiography (sampled at 200 Hz) and beat-to-beat systolic and diastolic blood pressure (SBP, DBP) via finger pulse photoplethysmography (Portapres^®^, TPD Biomedical Instrumentation, Amsterdam, the Netherlands) at the index or middle finger [9,13]. Before recording of biosignals, blood pressure (BP) was calibrated against the ipsilateral brachial artery BP [9,13]. Data of each recording was analyzed and digitized on a custom-designed data acquisition and analysis system (SUEmpathy™, SUESS Medizin-Technik GmbH, Aue, Germany) [9,13].

From 5 min time-series we extracted most stationary 2 min segments, manually corrected for artifacts and calculated mean values and standard deviation (SD) of above-mentioned biosignals.

In a next step, time-domain parameters of cardiovascular autonomic modulation were calculated: the square root of the mean squared differences in successive RRIs (RMSSDs) which is assumed to reflect vagal cardiac modulation [9,13,14]. Additionally, the coefficient of variation of RRIs (RRI-CV) as an absolute measure of dispersion and the RRI-SD as a relative measure of dispersion were calculated. Both parameters are known to reflect sympathetic and vagal cardiac modulation [9,13,14].

In a final step, we performed analysis of slow underlying RRI and BP oscillations that are largely mediated by undulating sympathetic and parasympathetic activity, using trigonometric regressive spectral analysis (TRS) [14]. The TRS algorithm identifies peaks within the low-frequency (LF; 0.04–0.14 Hz) and high-frequency (HF; 0.15–0.50 Hz) range of oscillations of RRI and BP modulation [13,14]. Calculated as an integral under the power spectral density curves, LF and HF oscillations of RRI (ms^2^/Hz) and BP (mmHg^2^/Hz) can be defined as LF and HF powers [13,14]. RRI-LF powers have been described to be influenced by sympathetic and, to an undetermined degree, by parasympathetic outflow, whereas SBP-LF powers exclusively depend upon sympathetic outflow [13,14]. Regarding HF oscillations, RRI-HF powers have been related to parasympathetic outflow, while BP-HF powers are supposed to be caused by respiratory-induced fluctuations in cardiac output and venous return [13,14]. Furthermore, we calculated total powers of RRI oscillations (RRI-total powers) as a sum of LF and HF powers, i.e., a parameter for total autonomic cardiac modulation, and RRI-LF/HF ratios, i.e., an index for sympathovagal balance [13,14]. Finally, to adjust for differences in overall signal modulation, we calculated normalized RRI-LF and HF powers (RRI-LF(nu) powers = [LF/(LF + HF)] × 100%, RRI-HF(nu) powers = [HF/(LF + HF)] × 100%) as percentage values in relation to RRI-total power in the entire range from 0.04 Hz to 0.5 Hz [13,14].

### 2.5. Statistical Analysis

A commercially available statistical program (IBM SPSS Statistics for Windows, version 25, Armonk, NY, United States of America) was used for data analyses. Significance level was set at *p* < 0.05. Using the Kolmogorov–Smirnov test, we tested data for normal distribution. Data are expressed as median values and interquartile range (IQR). For most analyses, we categorized patients according to the occurrence of DCI during the disease course into two groups: DCI and No DCI. For comparison of non-normally distributed data, the Mann–Whitney U test was used, and for normally distributed data, the independent T-test was used. To investigate associations between parameters of cardiovascular autonomic testing and sonography with development of DCI we performed receiver-operating-characteristic (ROC) analysis. In order to evaluate clinical implication, the SAH cohort was categorized according to functional outcome after 3 to 6 months, regardless of DCI in a final analysis. Therefore, we categorized patients into two groups, favorable outcome (mRS ≤ 3) and unfavorable outcome (mRS > 3), and again performed the above-mentioned tests for group comparisons.

## 3. Results

### 3.1. Baseline Characteristics

Over a 12-month period, 78 patients with SAH were admitted to the Department of Neurology of the University Hospital of Erlangen and screened for eligibility. A total of 53 patients were included in the study. Inclusion and exclusion criteria as well as subgroups of study participants (SBI, i.e., DCI, and functional outcome, i.e., mRS after 3 to 6 months) are summarized in Figure 1. Diagnosis of DCI was made upon CT imaging during hospital stay within days 4 to 14 after SAH onset, and occurred in 17 patients of the study cohort. Clinical baseline characteristics of patients are presented in Table 1 and Table 2 Comparing the two subgroups DCI vs. No DCI (Table 1), there were no significant differences regarding age, sex, prior comorbidities, premedication, clinical parameters and outcome parameters. Furthermore, radiological parameters at hospital admission were similar between groups; only the percentage of patients with concomitant ICH was higher in the DCI than in No DCI group (52.9 vs. 13.9%, *p* < 0.01). When comparing baseline values of the two ssubgroups favorable vs. unfavorable outcome (Table 2), we found no significant differences regarding age, sex, prior comorbidities, and premedication. Yet, clinical parameters at admission differed between groups, as patients with unfavorable outcome had significantly higher Hunt and Hess Scores (4 vs. 2, *p* < 0.01), higher NIHSS scores (38 vs. 0, *p* < 0.01), higher WFNS scores (5 vs. 1, *p* < 0.01), and higher rates of mechanical ventilation on day one (100 vs. 51.5%, *p* < 0.01) than patients with favorable outcome (Table 2). Again, radiological parameters at hospital admission were similar between groups, only the percentage of patients with concomitant ICH—especially within the left hemisphere—was higher in the unfavorable outcome than in favorable outcome group (60.0 vs. 15.2%, *p* < 0.01).

### 3.2. Comparison of Parameters Between Patients with and Without Delayed Cerebral Ischemia

#### 3.2.1. Transcranial Doppler Ultrasound

**Figure 2:**
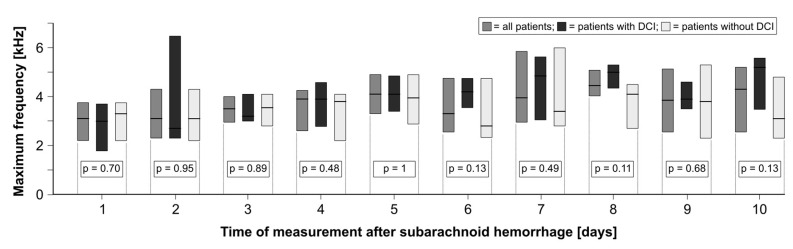
Maximum transcranial Doppler ultrasound frequencies from admission to day 10 of hospital stay. [*DCI*, delayed cerebral ischemia].

Overall, frequencies assessed using TCD did not differ significantly between patients with and without DCI (*p* > 0.05)—neither during the acute phase of SAH, i.e., within 24 h after bleeding, nor during consecutive assessments until day 10 (Figure 2; Table 3). Furthermore, the vascular territory, i.e., location of maximum frequencies, was similar in both groups (Table 3). TCD revealed maximum frequencies on day 10 within the DCI cohort, and on day 8 in patients without DCI.

[*DCI*, delayed cerebral ischemia; *Max*., maximum].

Until day 4, both groups had mean TCD values between normal and borderline. On day 5, mean TCD values increased above 4 kHz in DCI patients, with a further increase until the maximum values on day 10 (5.2 kHz). In No DCI patients, mean TCD values ranged between a minimum of 2.8 kHz (i.e., normal) on day 6, and a maximum of 4.1 kHz on day 8 (significant vasospasm).

#### 3.2.2. Biosignals

Within the acute phase of SAH, as well as during consecutive assessments until day 10, patients with and without DCI had comparable values regarding biosignals that were used for further analyses of autonomic cardiovascular modulation, i.e., R–R intervals (RRIs) and systolic blood pressure (SBP). For better illustration, Table 4 shows examples of the recorded biosignals and consecutively derived parameters of autonomic cardiovascular modulation within 24 h and on day 5 after SAH.

#### 3.2.3. Parameters of Autonomic Cardiovascular Modulation

**Figure 3:**
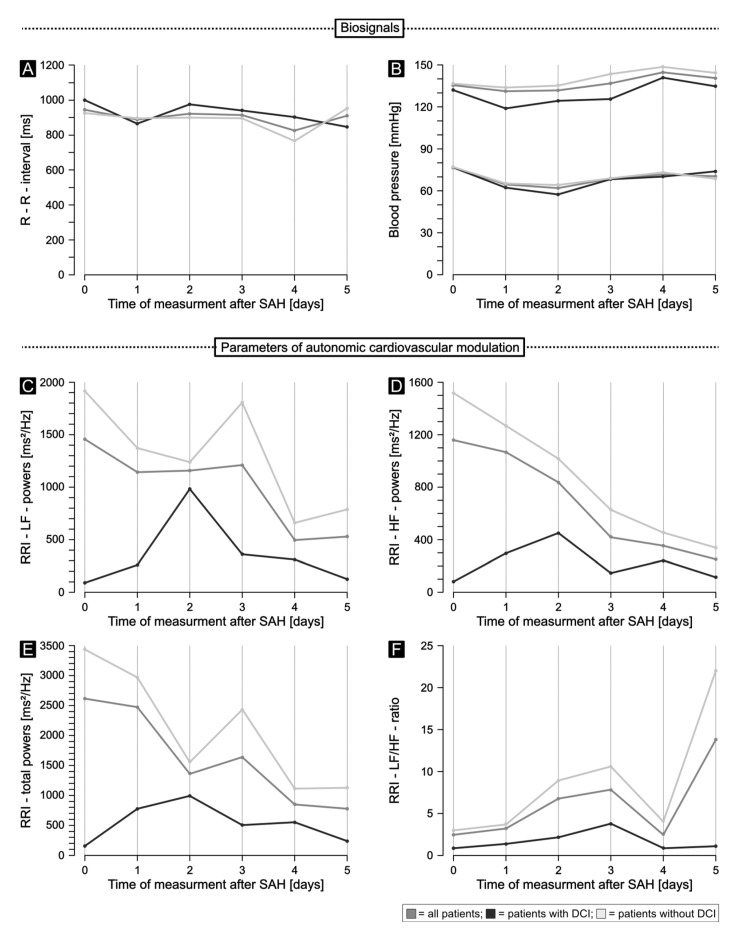
Biosignals and parameters of autonomic cardiovascular modulation, from admission to day 5 of hospital stay. [SAH, subarachnoid hemorrhage; DCI, delayed cerebral ischemia; LF, low frequency; HF, high frequency].

Figure 3 illustrates biosignals and parameters of autonomic cardiovascular modulation, during the first 5 days after SAH.

#### 3.2.4. Sympathetic Modulation

Compared to No DCI patients, our DCI cohort had significantly lower values of sympathetic modulation, i.e., RRI-LF powers and SBP-LF powers, on days 5 and 9 after SAH (RRI-LF powers: on day 5 9.3 vs. 587.3 ms^2^, *p* = 0.01, on day 9 38.2 vs. 336.4 ms^2^, *p* = 0.04; SBP-LF powers: on day 5 3.4 vs. 9.1 mmHg^2^, *p* = 0.04, on day 9 10.1 vs. 15.6 mmHg^2^, *p* = 0.04; Figure 3 and Table 4).

Already during the first 24 h, i.e., the first assessment, patients with DCI had an insignificant trend towards lower values reflecting sympathetic tone. In DCI patients, these values increased insignificantly on days 1 and 2, and were partly even slightly higher than in No DCI patients. On day 3, RRI and SBP-LF powers started to decrease again in DCI patients—to values lower than those of No DCI patients—reaching the above-mentioned significant differences on days 5 and 9. The additionally calculated value of normalized RRI-LF powers were insignificantly different in patients with and without DCI on days 5 and 9 after SAH, with lower values in the DCI cohort.

#### 3.2.5. Parasympathetic Modulation

Furthermore, without reaching statistical significance, our cohort of DCI patients had lower values of parasympathetic modulation, i.e., RMSSDs and RRI-HF powers, compared to No DCI patients on day 5 after SAH (RMSSD: 10.3 vs. 27.0 ms, *p* = 0.07; RRI-HF powers: 20.3 vs. 215.5 ms^2^, *p* = 0.07; Figure 3 and Table 4).

Similar to parameters reflecting sympathetic tone, patients with DCI had an insignificant trend towards lower values reflecting vagal outflow already during the first 24 h, i.e., the first assessment. In DCI patients, these values also increased insignificantly on days 1 and 2, and were partly even slightly higher than in No DCI patients. On day 3, RMSSDs and RRI-HF powers started to decrease again in DCI patients—to values lower than those of No DCI patients—reaching the above-mentioned differences on day 5. Compared to DCI patients, the cohort of No DCI patients had stable or even increasing values of parasympathetic modulation during the time course of assessment, with highest values on day 8 after SAH. Again, the additionally calculated value of normalized RRI-HF powers were insignificantly different in patients with and without DCI on days 5 and 9 after SAH, with higher values in the DCI cohort.

#### 3.2.6. Total Autonomic Modulation and Sympathovagal Balance

Finally, the above-mentioned trends during the disease course were consistently and reproducibly observed in parameters reflecting total autonomic modulation (RRI-SD, RRI-CV, RRI-total powers). Patients with DCI had an insignificant trend towards lower values already during the first 24 h, i.e., the first assessment. Again, in DCI patients, RRI-SD, RRI-CV, RRI-total powers increased insignificantly on days 1 and 2, and were partly even slightly higher than in No DCI patients. On day 3, parameters started to decrease again in DCI patients—to values significantly lower than those of No DCI patients on day 5 (RRI-SD: 9.6 vs. 34.1 ms, *p* = 0.01; RRI-CV: 1.0 vs 3.9%, *p* = 0.01; RRI-total powers: 29.5 vs. 1035.9 ms^2^, *p* < 0.01; Figure 3 and Table 4).

Compared to parameters of sympathetic, parasympathetic, and total autonomic modulation, RRI-LF/HF ratios, i.e., an index of sympathovagal balance, were significantly lower in DCI patients compared to No DCI patients only on day 9 after SAH (0.7 vs. 2.9, *p* < 0.01). Despite assessments on days 1 and 8, DCI patients had slightly, yet insignificantly lower LF/HF ratios than No DCI patients, without a clear trend similar to the above-mentioned findings of autonomic cardiovascular modulation.

### 3.3. Potential Influence of the Therapeutic Regimen on Parameters of Autonomic Cardiovascular Modulation

Given the observed findings of significant differences in cardiovascular autonomic modulation between DCI and No DCI patients on day 5 after SAH onset, we compared therapeutic regimens of both groups on this specific day of hospital stay (Table 5). Similar to absent differences in premedication upon hospital admission, percentage numbers of patients receiving nimodipine, vasopressor therapy, antihypertensive drugs, analgesic drugs, sedative drugs, or mechanical ventilation on day 5 did not differ between DCI and No DCI patients (for the complete list see Table 5).

Furthermore, to account for potential influences of applied therapies during ICU treatment on parameters of autonomic cardiovascular modulation, we compared differences in rescue strategies between DCI and No DCI patients (Table 6). During the course of the disease, numbers of patients receiving cerebrospinal fluid drainage (external ventricular drain or lumbar drain), and intraventricular thrombolysis were similar between both groups (Table 6). Yet, compared to the No DCI cohort, higher numbers of DCI patients received intra-arterial vasospasmolysis, and osmotic active therapy (mannitol and/or hypertonic saline) throughout their hospital stay (Table 6).

### 3.4. Associations Between Parameters of Cardiovascular Autonomic Testing and Transcranial Doppler Ultrasound with Development of DCI

Due to the observed differences in autonomic cardiovascular modulation between patients with and without DCI, reaching statistical significance on day 5 after SAH, we calculated delta values (i.e., value on day 5 minus value of first assessment; delta_5-0) for each parameter in both patient groups.

Despite decreases in all assessed parameters of autonomic modulation in DCI patients until day 5, comparison of delta_5-0 values between patients with and without DCI did not reveal significant levels for any autonomic parameter. This finding translates into statistical association analyses; no significant associations between delta_5-0 values and the occurrence of DCI were observed.

Similar to the findings in absolute TCD values in patients with and without DCI during the time course of assessments, TCD delta_5-0 was similar in both patient groups, without significant associations to discriminate for occurrence of DCI (AUC: 0.69).

### 3.5. Associations Between Parameters of Cardiovascular Autonomic Testing and Transcranial Doppler Ultrasound with Functional Outcome

In order to validate clinical implication and importance of the observed findings, our SAH cohort was categorized according to functional outcome after 3 to 6 months in a final step (Table 2). Dichotomization into the two groups, favorable (mRS ≤ 3; 33 patients) vs. unfavorable (mRS > 3; 15 patients) outcome (regardless of DCI), revealed the following findings on day 5 after SAH (Table 7): Parameters of sympathetic modulation (RRI-LF powers, SBP-LF powers), parameters of parasympathetic modulation (RRI-HF powers, RMSSDs), and parameters of total autonomic modulation (RRI-LF/HF-ratios, RRI-SD, RRI-CV) did not differ significantly between patients with favorable and unfavorable outcome, but showed slightly lower values in the unfavorable outcome group. Yet, the additionally calculated values of normalized RRI-LF powers (0.6 vs. 0.4, *p* = 0.03) and normalized RRI-HF powers (0.4 vs. 0.6, *p* = 0.03), as well as LF/HF ratios (1.5 vs. 0.7, *p* = 0.03) were significantly different in patients with and without favorable outcome (Table 7). This finding again suggests a significant association between the above-mentioned reduction of sympathetic and parasympathetic modulation on day 5 after SAH and a poor clinical outcome.

## 4. Discussion

### 4.1. Summary of Key Findings

The present study yielded the following relevant findings:

1. Not only during the acute phase, i.e., within 24 h [9], but also during the first days after disease onset, spontaneous SAH induces a decrease in sympathetic, parasympathetic and total autonomic cardiovascular modulation.
2. Significantly lower values of sympathetic modulation (RRI-LF powers, normalized RRI-LF powers, SBP-LF powers) on days 5 and 9 and significantly lower values of total autonomic modulation (RRI-SD, RRI-CV, RRI-total powers), as well as insignificantly lower values of parasympathetic modulation (RMSSDs, RRI-HF powers) on day 5 after SAH in those patients who develop DCI compared to the No DCI cohort, suggest a clear attribution of autonomic mechanisms on cerebral macro and microvasospasms and finally secondary brain injury.
3. Regardless of DCI development, significantly different normalized RRI-LF powers and normalized RRI-HF powers, as well as LF/HF ratios on day 5 in patients with mRS > 3 compared to the mRS ≤ 3 cohort three to six months after SAH, revealed potential associations of autonomic dysfunction on clinical outcome. Thus, bedsided assessment of heart rate, and blood-pressure variability may predict augmented risk of cardiovascular complications.
4. In contrast, despite expected increases in TCD frequencies during the disease course, this standard diagnostic evaluation for detecting clinically relevant vasospasms did not reveal significant associations with DCI development in our cohort.
5. Finally, although the interpretability may be limited by the small sample size, absent differences in premedication as well as the therapeutic regimen on day 5 indicate that potential iatrogenic confounders may not account for the observed autonomic dysregulation in SAH patients who develop DCI.

### 4.2. Autonomic Dysfunction, Development of DCI, and Functional Outcome—Pathophysiological Context

According to the recent literature, several studies have demonstrated associations between impaired autonomic modulation after SAH and increased incidences of secondary medical events, both primary neurological as well as extracerebral complications. Furthermore, correlations between autonomic dysfunction and poor functional outcomes and an elevated mortality risk have not only been described in patients after SAH but also other stroke subtypes and various critical care conditions [8,9,15]. Within the literature, autonomic dysregulation is commonly described as altered heart rate variability (HRV), with most studies reporting reduced cardiovascular modulation. However, despite differences in methodological approaches, the aforementioned studies reported markedly divergent findings regarding both the cause and extent of autonomic dysregulation and sympathovagal balance. While some authors proposed a pronounced sympathetic predominance, others describe augmented parasympathetic tone [8]. These inconsistencies are likely attributable, at least in part, to a lack of standardization, particularly with respect to measurement timing. Notably, the paucity of investigations during the critical acute phase limits the ability to draw definitive conclusions regarding early autonomic impairment, rendering such interpretations largely speculative [15]. Furthermore, variability in reported findings may also be ascribed to the assessment of poorly comparable autonomic parameters [15].

Preliminary findings of our study cohort published in 2022 addressed several of these limitations [9]: in a structured analysis of HRV, including both time- and frequency-domain parameters that were obtained within 24 h after SAH, a significant reduction in total autonomic activity, as well as sympathetic cardiovascular modulation, was observed during the acute phase of the disease. These alterations were clearly associated with poor clinical outcome. Moreover, a significant negative correlation was found between altered autonomic modulation and increasing Hunt and Hess scores, i.e., clinical severity upon hospital admission [9]. In the present analysis of daily standardized measurements in the same patient cohort, this pattern of autonomic dysfunction was confirmed, both in patients who developed radiologically confirmed DCI as well as in patients with poor functional outcomes, as assessed by the mRS 3-– 6 months after hospital discharge (Table 7). Notably, day 5 after disease onset—corresponding to the period of expected cerebral vasospasm and DCI occurrence—appears to be particularly relevant, as our results suggest most pronounced impairment of autonomic function on this specific day of assessment with a significantly augmented decrease in sympathetic and vagal modulation compared to our No DCI subgroup (Table 7). Notably, there was no significant difference between the two DCI subgroups in terms of the use of vasopressors and analgosedation, which are known to be significant factors influencing the results of autonomic testing (Table 5).

Although early alterations in autonomic modulation (e.g., reduced HRV) during the initial days following SAH have been previously reported, the underlying pathophysiological mechanisms remain largely speculative [8]. One possible explanation for the early and potentially progressive autonomic dysfunction observed up to day 5 involves distinct disease-related processes. Muraoka et al. described an initial hyperadrenergic crisis with substantial catecholamine release during the acute phase of SAH compared to episodes of paroxysmal sympathetic hyperactivity, typically occurring around day 5 after disease onset [16]. These pathophysiologically different alterations may partly explain our findings and need be recognized in routine clinical practice as they have important implications for therapeutic strategies [17]. In addition to sympathetic changes, impairment of parasympathetic modulation has been reported after SAH [18]. A reduced vagal tone during the acute phase has been associated with the occurrence of the so-called “brain-neurocardiac web syndrome,” i.e., a SAH-induced degeneration of parasympathetic networks, including those within the cardiac plexus [18]. Again, this sequalae may also contribute to an increased risk of secondary cardiac complications [18]. In line with these results, our data suggest alterations of both branches of the autonomic cardiovascular modulation—with significant associations between reduced sympathetic but also vagal tone on day 5 and the development of DCI as well as an unfavorable outcome up to 6 months after SAH (Table 3 and Table 7). In addition, several other mechanisms underlying autonomic dysfunction after SAH have been proposed. For example, a reduced baroreflex sensitivity has been associated with prolonged regional cerebral desaturation, as measured using near-infrared spectroscopy (NIRS) [19]. Furthermore, other studies have suggested associations between diminished baroreflex sensitivity and impaired cerebral autoregulation [19].

Despite the lack of definitive pathophysiological explanations, there is general consensus that reduced HRV following SAH correlates with poor clinical outcome. This aspect was demonstrated not only within the acute phase (as shown by the 2022 study), but also in the present analysis using repeated and daily measurements after hospitalization; Table 7 illustrates these clear associations between dysfunction of autonomic modulation and long-term outcome. The concept of the so-called brain–heart axis provides a plausible explanatory framework, describing the close interaction between central autonomic network structures and cardiac autonomic regulation in the context of both early and secondary brain injury [20]. In this model, excessive catecholamine release is thought to exert direct myocardial effects, leading to a spectrum of electrocardiographic changes ranging from benign alterations to life-threatening arrhythmias and severe non-ischemic cardiomyopathy [21]. Moreover, Megihani et al. suggested that the finding of an increased sympathovagal balance, i.e., elevated RRI LF/HF ratio within the first 48 h after SAH, may be associated with an augmented risk of cardiac complications, including motion abnormalities, ventricular dysfunction, and troponin elevation [22]. In line with our findings of significant autonomic impairment, particularly on day 5 after SAH (Table 6), that precedes the occurrence of DCI and is associated with poor functional outcomes after 3–6 months, Megihani et al. suggested that pathological HRV alterations may occur even before SBI may develop [22]. In fact, our data demonstrate that these alterations may occur already upon disease onset and persistently deteriorate during the first days after SAH, with a peak in autonomic dysregulation on day 5 (Figure 3).

Importantly, autonomic dysfunction appears to be relevant not only for SBI but may also predict long-term outcomes. In addition to the previous findings of our group [9], i.e., association between early autonomic cardiovascular dysregulation and poor functional status at hospital discharge (mRS), Wenneberg et al. demonstrated worse functional outcomes even up to one year after disease onset in patients with sympathovagal imbalance during the first two days after SAH [23]. In line with these results, we were able to demonstrate clear associations between early autonomic dysfunction and poor SAH outcome after 3–6 months, i.e., significantly different normalized RRI-LF and HF powers in patients with mRS > 3 (Table 7).

In summary, HRV analysis after SAH appears to represent a promising tool for risk stratification regarding both secondary neurological as well as extracerebral complications. Thus, identification of patients at risk may enable physicians to implement targeted therapeutic and preventive strategies in daily practice. Moreover, persistent and clinically relevant autonomic dysfunction may serve as a predictor of poor functional outcome and therefore potentially influence decision making. Yet, further studies are needed to validate reliability and clinical applicability of this approach. Potential future directions include the use of continuous HRV monitoring [23,24] as well as the integration of modern machine learning algorithms [25].

### 4.3. TCD—Recommended Monitoring Technique with Tenuous Benefits

DCI, as a key contributor to the development of SBI after SAH, is clearly associated with survival as well as long-term functional outcomes—and can be detected using various neuromonitoring modalities according to international guidelines [4,26]. Across recommendations, routine TCD monitoring represents the standard of care diagnostic tool [4,26], although clear associations with the occurrence of DCI have not been well established [27]. Despite an acceptable specificity of approximately 71% (95% CI, 51–85%) [4], reported sensitivities range below 40% [26], thus substantially limiting the predictive value of TCD for the detection of clinically relevant vasospasm. Consistent with these limitations, our data did not demonstrate significant differences in maximum TCD velocities between patients with and without DCI. Although TCD values technically met the criteria of “significant vasospasm” starting on day 5 after SAH in patients who developed DCI, and the expected overall increase during the course of hospitalization was observed (Figure 2), our findings further underscore the limited utility of this monitoring modality in distinguishing between patients at risk and those not at risk of developing DCI, and consequently SBI.

Several studies suggested that the predictive performance of TCD may be improved when considering specific conditions. In particular, the timing of TCD measurements after the hemorrhagic event appears to be relevant in this context. Kelly et al. demonstrated that the absence of Doppler-detectable vasospasm until day 10 after symptom onset is associated with a high probability (approximately 90%) of not developing vasospasm during the subsequent, entire course of disease [28]. Moreover, mild vasospasm on days 4–5 and moderate vasospasm on days 6–9 after SAH have been associated with radiologically confirmed vasospasm [27]. However, despite an acceptable negative predictive value (>90%), a positive predictive value of approximately 20% again highlights the limited sensitivity of this approach [7]. In addition, specific clinical and radiological parameters, e.g., high modified Fisher scale score, elevated Hunt and Hess (H&H) grade, age < 50 years at admission, and increased initial SBP have also been suggested helpful predictors for early identification of patients at risk of developing DCI [27,28]. In contrast, our cohort did not demonstrate significant differences in baseline characteristics between patients with and without DCI, neither with respect to clinical nor radiological parameters (Table 1). Only the presence of an initial intracerebral hemorrhage was significantly more frequent in patients who developed DCI.

The complex pathophysiology of cerebral macro- and microvasospasm, from which the multifactorial etiology of DCI is derived, may explain the heterogeneous findings and the limited sensitivity of TCD and other parameters for detecting SBI [29]. Contributing factors to cerebral vasospasm and DCI include the volume of subarachnoid blood and its distribution within the CSF [30], the concentration of hemoglobin in the CSF with a resulting adaptive macrophage response [31], and, for example, iron deposition within white matter regions [32]. In addition, inflammatory mechanisms (e.g., local microglial activation) [33] and catastrophic ionic disturbances known as cortical spreading depolarizations, which in turn trigger neuroinflammatory processes, microthrombosis, and cerebral vasoconstriction, contribute to the development of vasospasm and DCI [34].

Accordingly, there is a clear need for additional monitoring modalities. Although not yet sufficiently validated, within the literature several approaches are described: CT angiography (with sensitivities up to 97%) for the detection of macrovascular spasm; CT perfusion for identifying reduced cerebral perfusion—even in the absence of angiographic vasospasm; and digital subtraction angiography (DSA) as the gold standard [26]. Further modalities include continuous EEG monitoring as well as invasive neuromonitoring techniques such as PbtO_2_ measurement, microdialysis for lactate/pyruvate ratios and glutamate levels, electrocorticography (ECoG), and intracortical EEG [4,26]. However, beyond concerns regarding patient safety, studies on invasive neuromonitoring strategies are subject to various sources of bias, including patient selection, lack of standardized timing of monitoring initiation, and outcome ascertainment [4]. Consequently, aside from regularly assessed TCD, none of these modalities have yet been incorporated into firm guideline recommendations.

In light of these findings, autonomic function testing following SAH may represent a promising additional component within a multimodal neuromonitoring framework.

### 4.4. Therapeutic Regimen, Rescue Strategies, Clinical Outcome, and Future Directions

Even though associations between different classes of medication and cardiovascular autonomic modulation are well known, we did not observe differences in premedication (Table 1) or the therapeutic regimen on day 5 (Table 4) between patients with and without DCI after SAH. Consequently, potential iatrogenic confounders may not account for the observed autonomic dysregulation in SAH patients who develop DCI.

Despite declining incidences of SAH on the one hand and substantial developments and improvements in the management of EBI on the other hand, detection and therapy of SBI remains object to further research. For decades, the gold standard for the prophylaxis of cerebral vasospasm and associated DCI has remained unchanged and comprises the administration of nimodipine [1]. Accordingly, 100% of our patients of both cohorts received this specific treatment (Table 4). During recent years, one study stood out in particular, demonstrating significant advantages in functional outcome with the use of lumbar CSF drainage after SAH (EARLY-DRAIN) [35]. These results were supported by a meta-analysis of additional RCTs evaluating LD and functional outcome 6 months after disease onset [36]. With regard to the use of CSF drainage (via EVD or LD), no significant differences were observed in our two cohorts of patients with and without DCI after SAH (Table 5). Consequently, we again suggest a clear attribution of the observed autonomic dysregulation to cerebral macro- and microvasospasms, and ultimately SBI.

Further potential therapeutic options in the prophylaxis of SBI are currently under investigation. In the context of autonomic modulation, recent data on electrical stimulation (spinal cord stimulation, stimulation of the sphenopalatine ganglion) [37,38,39], as well as the use of renal denervation for reduction of cerebral vasospasm and stabilization of sympathetic cardiovascular modulation [40], have been published with promising results. So far, the insufficient body of evidence argues against a general therapeutic use of these strategies in SAH patients. Yet—considering our findings—a combination of improved detection of patients at risk of developing DCI/SBI using autonomic function testing and implementation of therapeutic strategies, particularly targeting dysregulation of the autonomic nervous system, would be desirable and should be the subject of future investigations.

### 4.5. Limitations

Despite our interesting findings, which describe associations between the severity of autonomic dysfunction in patients with spontaneous subarachnoid hemorrhage and the occurrence of DCI as well as long-term outcomes, several important limitations remain. First and foremost, due to the small observational cohort it is not possible to identify clear effects of autonomic dysfunction or to deduce predictions regarding key clinical parameters; ultimately, our results can only be integrated as associations. Furthermore, due to the small subgroups and the relatively large number of confounders (e.g., disease severity, mechanical ventilation, sedation depth, vasopressor dose, intracranial hemorrhage, rescue therapies, etc.), we were not able to carry out statistically meaningful multivariate analyses. Despite improving statistical validity, the fact has to be highlighted that results remain exploratory without correction in terms of mentioned biases. Moreover, since only patients who were identified as having DCI based on radiological findings (i.e., new cerebral infarction on CT) yet not based on clinical findings (i.e., deterioration of clinical presentation including a difference of 2 points on the NIHSS or the Glasgow Coma Scale, possibly supported by screening tools as VASOGRADE) were included in the “DCI” subgroup, no conclusions can be drawn regarding this patient cohort, which is clearly not less important to the team of treating physicians in everyday clinical practice. None of our patients had radiographic evidence of a cerebral infarction as early as day 5, i.e., within the time window that appeared to be relevant for dysregulation of autonomic cardiovascular modulation in our cohort. Once again, the small sample size limits interpretability of the results—in this context, since a possible temporal relationship between autonomic decline and DCI itself is not reflected in these findings. One possible approach for follow-up studies appears to be a patient-level timeline specifically analyzing the onset and detection of DCI relative to HRV changes and therapeutic regimens. Finally, the study is likely underpowered to prove whether the use of HRV testing is truly superior to bedside TCD monitoring. Final conclusions need to be determined in larger study populations, ideally through a multi-center setting.

### 4.6. Conclusions

In conclusion, our data demonstrate clear associations between an early impairment of autonomic cardiovascular modulation within the first days after SAH onset and the development of secondary brain injury, i.e., DCI, and also an unfavorable outcome up to 6 months after disease onset. No comparable associations were identified using recommended TCD, suggesting that bedside HRV testing may represent a promising, noninvasive adjunctive marker within multimodal neuromonitoring of critically ill patients following spontaneous SAH. In summary, HRV testing is promising but not yet reliable without external validation. Further research in this area is needed to validate our findings and indicate potential influences on decision making in clinical practice.

## Figures and Tables

**Table 1:** Baseline characteristics of 53 patients with spontaneous subarachnoid hemorrhage and subgroups regarding secondary brain injury.

	All Patients (*n* = 53)	DCI (*n* = 17)	No DCI (*n* = 36)	*p*-Values (*DCI vs. No DCI*)
**Age** [years], Median (IQR)	54 (49.0–62.5)	54 (49.5–66.5)	53 (48.5–60.8)	0.36
**Female sex**, n (%)	28 (52.8)	8 (47.1)	20 (55.6)	0.78
**Prior comorbidities**				
Arterial hypertension, n (%)	28 (52.8)	12 (70.6)	16 (44.4)	0.14
Diabetes mellitus, n (%)	8 (15.1)	3 (17.6)	5 (13.9)	1
Dyslipidemia, n (%)	8 (15.1)	3 (17.6)	5 (13.9)	1
Prior ischemic stroke/TIA, n (%)	1 (1.9)	1 (5.9)	0 (0.0)	0.32
Prior hemorrhagic stroke, n (%)	0 (0.0)	0 (0.0)	0 (0.0)	1
Congestive heart failure, n (%)	4 (7.5)	2 (11.8)	2 (5.6)	0.59
**Premedication**				
Antihypertensive drugs, n (%)	18 (34.0)	6 (35.3)	12 (33.3)	0.86
- ACE inhibitor/ARB, n (%)	14 (26.4)	4 (23.5)	10 (27.8)	1
- Betablocker, n (%)	7 (13.2)	2 (11.8)	5 (13.9)	1
- Diuretic, n (%)	11 (20.8)	4 (23.5)	7 (19.4)	1
- Calcium channel blocker, n (%)	3 (5.7)	1 (5.9)	2 (5.6)	1
Antiplatelet drug, n (%)	2 (3.8)	1 (5.9)	1 (2.8)	1
Anticoagulation, n (%)	7 (13.2)	3 (17.6)	4 (11.1)	0.67
**Clinical parameters**				
Hunt & Hess score, Median (IQR)	3 (1–4)	4 (2–4)	3 (1–4)	0.43
NIHSS, Median (IQR)	12 (0–38)	38 (0–38)	12 (0–38)	0.35
WFNS scale, Median (IQR)	3 (1–5)	4 (1–5)	3 (1–5)	0.41
Pre-mRS, Median (IQR)	0 (0–0)	0 (0–0)	0 (0–0)	0.97
Mechanical ventilation on day 1, n (%)	34 (64.2)	13 (76.5)	21 (58.3)	0.24
**Radiological parameters**				
mFS, Median (IQR)	4 (3–4)	4 (3–4)	4 (3–4)	0.59
Cerebral aneurysm, n (%)	39 (73.6)	14 (82.4)	25 (69.4)	0.51
- Anterior circulation, n (%)	28 (52.8)	12 (70.6)	16 (44.4)	0.14
- Posterior circulation, n (%)	11 (20.8)	5 (29.4)	6 (16.7)	0.47
Intracerebral hemorrhage, n (%)	14 (26.4)	9 (52.9)	5 (13.9)	* <0.01
- Right hemisphere, n (%)	5 (9.4)	3 (17.6)	2 (5.6)	0.31
- Left hemisphere, n (%)	8 (15.1)	5 (29.4)	3 (8.3)	0.09
- Corpus callosum, n (%)	1 (1.9)	1 (5.9)	0 (0.0)	0.32
Intraventricular hemorrhage, n (%)	30 (56.6)	9 (52,9)	21 (58.3)	0.77
Acute occluding hydrocephalus, n (%)	32 (60.4)	11 (64.7)	21 (58.3)	0.88
**Outcome parameters**				
LOS [days], Median (IQR)	17 (11–23)	17 (12.5–23.5)	17 (10.3–23.0)	0.98
In-hospital death, n (%)	6 (11.3)	2 (11.8)	4 (11.1)	1
mRS after 1 to 3 months, Median (IQR)	1 (1–4)	3 (1–5)	1 (1–4)	0.52
mRS after 3 to 6 months, Median (IQR)	1 (0–4)	1 (0–6)	1 (0–3)	0.26
mRS after 3 to 9 months, Median (IQR)	1 (0–3)	1 (0–5.3)	1 (0–3)	0.44

[*DCI*, delayed cerebral ischemia; *TIA*, transient ischemic attack; *ACE*, angiotensin-converting enzyme; *ARB*, angiotensin II receptor blockers; *WFNS*, World Federation of Neurosurgical Societies; *NIHSS*, National Institutes of Health Stroke Scale; *mRS*, modified Rankin Scale; *mFS*, modified Fisher scale; *LOS*, length of stay]. * and bold indicate significant differences.

**Table 2:** Baseline characteristics of patients with spontaneous subarachnoid hemorrhage and subgroups regarding functional outcome.

	All Patients (*n* = 53)	Favorable Outcome (mRS ≤ 3; *n* = 33)	Unfavorable Outcome (mRS > 3; *n =* 15)	*p*-Values (*mRS* ≤ 3 *vs. mRS >* 3)
**Age** [years], Median (IQR)	54.0 (50.0–62.8)	53.0 (47.0–62.0)	57.0 (53.0–66.0)	0.09
**Female sex**, n (%)	25 (52.1)	18 (54.5)	7 (46.7)	0.84
**Prior comorbidities**				
Arterial hypertension, n (%)	25 (52.1)	16 (48.5)	9 (60.0)	0.67
Diabetes mellitus, n (%)	7 (14.6)	5 (15.2)	2 (13.3)	1
Dyslipidemia, n (%)	8 (16.7)	6 (18.2)	2 (13.3)	1
Prior ischemic stroke/TIA, n (%)	1 (2.1)	0	1 (6.7)	0.31
Prior hemorrhagic stroke, n (%)	0	0	0	1
Congestive heart failure, n (%)	4 (8.3)	2 (6.1)	2 (13.3)	0.58
**Premedication**				
Antihypertensive drugs, n (%)	16 (33.3)	8 (24.2)	8 (53.3)	0.10
- ACE inhibitor/ARB, n (%)	12 (25)	8 (24.2)	4 (26.7)	1
- Betablocker, n (%)	7 (14.6)	6 (18.2)	1 (6.7)	0.41
- Diuretic, n (%)	9 (18.8)	4 (12.1)	5 (33.3)	0.11
- Calcium channel blocker, n (%)	3 (6.3)	2 (6.1)	1 (6.7)	1
Antiplatelet drug, n (%)	2 (4.2)	1 (3.0)	1 (6.7)	1
Anticoagulation, n (%)	6 (12.5)	4 (12.1)	2 (13.3)	1
**Clinical parameters**				
Hunt & Hess score, Median (IQR)	3 (1–4)	2 (1–4)	4 (3–5)	* <0.01
NIHSS, Median (IQR)	2 (0–38)	0 (0–3)	38 (38)	* <0.01
WFNS scale, Median (IQR)	3 (1–5)	1 (1–4)	5 (4–5)	* <0.01
Pre-mRS, Median (IQR)	0 (0–0)	0 (0–0)	0 (0–0)	0.14
Mechanical ventilation on day 1, n (%)	32 (66.7)	17 (51.5)	15 (100)	* <0.01
**Radiological parameters**				
mFS, Median (IQR)	4 (3–4)	4 (3–4)	4 (3–4)	0.16
Cerebral aneurysm, n (%)	37 (77.1)	26 (78.8)	11 (73.3)	0.72
- Anterior circulation, n (%)	28 (52.8)	19 (57.6)	8 (53.3)	1
- Posterior circulation, n (%)	11 (20.8)	7 (21.2)	3 (20.0)	1
Intracerebral hemorrhage, n (%)	14 (29.2)	5 (15.2)	9 (60.0)	* <0.01
- Right hemisphere, n (%)	5 (9.4)	2 (6.1)	2 (13.3)	0.58
- Left hemisphere, n (%)	8 (15.1)	2 (6.1)	6 (40.0)	* <0.01
- Corpus callosum, n (%)	1 (1.9)	1 (3.0)	0	1
Intraventricular hemorrhage, n (%)	27 (56.3)	20 (60.6)	7 (46.7)	0.55
Acute occluding hydrocephalus, n (%)	29 (60.4)	17 (51.5)	12 (80.0)	0.11
DCI, n (%)	15 (31.3)	10 (30.3)	5 (33.3)	0.73
**Outcome parameters**				
LOS [days], Median (IQR)	18 (11–23)	18 (11–24)	16 (4–23)	0.56
In-hospital death, n (%)	6 (12.5)	0	6 (40.0)	* <0.01

[*DCI*, delayed cerebral ischemia; *TIA*, transient ischemic attack; *ACE*, angiotensin-converting enzyme; *ARB*, angiotensin II receptor blockers; *WFNS*, World Federation of Neurosurgical Societies; *NIHSS*, National Institutes of Health Stroke Scale; *mRS*, modified Rankin Scale; *mFS*, modified Fisher scale; *LOS*, length of stay]. * and bold indicate significant differences.

**Table 3:** Associations between transcranial Doppler ultrasound data and development of delayed cerebral ischemia.

	All Patients (*n* = 53)	DCI (*n* = 17)	No DCI (*n* = 36)	*p*-Values (*DCI vs. No DCI*)
**Max. frequencies day 1**, Median (IQR)	3.10 (2.20–3.75)	3.00 (1.78–3.70)	3.30 (2.20–3.75)	0.70
**Max. frequencies day 2**, Median (IQR)	3.10 (2.30–4.30)	2.70 (2.30–6.48)	3.10 (2.20–4.30)	0.95
**Max. frequencies day 3**, Median (IQR)	3.50 (2.95–4.00)	3.20 (3.00–4.10)	3.55 (2.80–4.08)	0.89
**Max. frequencies day 4**, Median (IQR)	3.90 (2.60–4.25)	3.90 (2.78–4.58)	3.80 (2.20–4.10)	0.45
**Max. frequencies day 5**, Median (IQR)	4.10 (3.30–4.90)	4.10 (3.40–4.85)	3.95 (2.88–4.90)	1
**Max. frequencies day 6**, Median (IQR)	3.30 (2.55–4.75)	4.20 (3.55–4.75)	2.80 (2.33–4.75)	0.13
**Max. frequencies day 7**, Median (IQR)	3.95 (2.95–5.85)	4.85 (3.05–5.63)	3.40 (2.80–6.00)	0.49
**Max. frequencies day 8**, Median (IQR)	4.45 (4.03–5.08)	5.00 (4.35–5.30)	4.10 (2.70–4.50)	0.11
**Max. frequencies day 9**, Median (IQR)	3.85 (2.55–5.13)	3.90 (3.50–4.60)	3.80 (2.30–5.30)	0.68
**Max. frequencies day 10**, Median (IQR)	4.30 (2.48–5.20)	5.20 (3.48–5.58)	3.10 (2.30–4.80)	0.13
**Location of max. frequencies**, n (%)				
Anterior cerebral artery	11 (20.8)	4 (23.5)	7 (19.4)	0.60
Middle cerebral artery	22 (41.5)	10 (58.8)	12 (33.3)	0.18
Carotid siphon	12 (22.6)	2 (11.8)	10 (27.8)	0.11
Posterior cerebral artery	0	0	0	1
Basilar artery	2 (3.8)	1 (5.9)	1 (2.8)	0.59
Vertebral artery	1 (1.9)	0	1 (2.8)	0.65

**Table 4:** Associations between biosignals as well as parameters of autonomic cardiovascular modulation and development of delayed cerebral ischemia within 24 h and on day 5 after spontaneous subarachnoid hemorrhage.

	All Patients (*n* = 53)	DCI (*n* = 17)	No DCI (*n* = 36)	*p*-Values (*DCI vs. No DCI*)
**DAY 1/INITIAL ASSESSMENT (within 24 h)**				
**Biosignals, median (IQR)**				
Systolic blood pressure [mmHg]	132.6 (116.5–147.1)	116.8 (109.3–138.3)	135.1 (125.3–148.5)	0.17
Diastolic blood pressure [mmHg]	66.1 (58.4–73.3)	63.0 (58.7–70.4)	66.6 (55.6–75.8)	0.72
Heart rate [min^−1^]	940.1 (765.8–1054.3)	932.6 (754.1–1040.9)	944.0 (758.9–1067.8)	0.84
**Sympathetic modulation**, median (IQR)				
RRI-LF powers [ms^2^]	163.6 (15.6–893.6)	163.6 (14.3–338.6)	156.8 (17.1–1328.2)	0.65
RRI-LF(nu) powers	0.5 (0.4–0.7)	0.4 (0.4–0.6)	0.6 (0.4–0.8)	0.15
SBP-LF powers [mmHg^2^]	4.6 (1.3–15.7)	2.9 (1.2–19.6)	4.8 (1.3–26.3)	0.91
**Parasympathetic modulation**, median (IQR)				
RRI-RMSSD [ms]	19.3 (8.9–47.8)	26.1 (17.9–38.2)	15.6 (7.8–60.1)	0.34
RRI-HF powers [ms^2^]	80.4 (17.5–429.0)	157.6 (36.5–282.4)	79.0 (17.4–1219.8)	0.77
RRI-HF(nu) powers	0.5 (0.3–0.6)	0.6 (0.4–0.7)	0.5 (0.2–0.6)	0.15
**Total autonomic modulation**, median (IQR)				
RRI-SD [ms]	23.0 (10.6–43.6)	24.3 (14.3–31.5)	20.2 (9.9–50.6)	0.84
RRI-CV [%]	2.4 (1.2–5.2)	3.0 (1.4–4.0)	2.2 (1.2–5.9)	0.87
RRI-total powers [ms^2^]	237.3 (66.5–1458.8)	360.2 (57.7–713.7)	232.6 (60.9–2665.1)	0.91
**Sympathovagal balance**, median (IQR)				
RRI-LF/HF ratio	1.5 (0.8–2.5)	0.9 (0.4–2.0)	1.6 (0.9–3.5)	0.20
**DAY 5**				
**Biosignals, median (IQR)**				
Systolic blood pressure [mmHg]	138.8 (132.2–149.9)	132.6 (125.5–144.8)	141.4 (135.4–150.5)	0.12
Diastolic blood pressure [mmHg]	70.2 (61.4–75.5)	70.2 (57.7–77.2)	70.9 (65.3–74.8)	1
Heart rate [min^−1^]	882.2 (744.3–1030.6)	829.6 (717.8–1006.2)	883.7 (740.3–1096.1)	0.52
**Sympathetic modulation**, median (IQR)				
RRI-LF powers [ms^2^]	207.1 (8.4–989.7)	9.3 (3.8–278.8)	587.3 (87.9–1210.8)	* 0.01
RRI-LF(nu) powers	0.6 (0.4–0.7)	0.5 (0.3–0.6)	0.6 (0.5–0.7)	0.09
SBP-LF powers [mmHg^2^]	5.9 (3.3–18.0)	3.4 (1.9–9.6)	9.1 (4.5–51.5)	* 0.04
**Parasympathetic modulation**, median (IQR)				
RRI-RMSSD [ms]	18.9 (6.1–37.8)	10.3 (4.6–19.6)	27.0 (11.0–40.3)	0.07
RRI-HF powers [ms^2^]	83.3 (7.0–575.8)	20.3 (6.2–145.3)	215.5 (28.3–612.5)	0.07
RRI-HF(nu) powers	0.4 (0.3–0.6)	0.5 (0.4–0.7)	0.4 (0.3–0.5)	0.09
**Total autonomic modulation**, median (IQR)				
RRI-SD [ms]	18.0 (4.9–46.8)	9.6 (3.7–24.2)	34.1 (14.7–53.6)	* 0.01
RRI-CV [%]	1.7 (0.6–5.3)	1.0 (0.4–0.5)	3.9 (1.2–5.6)	* 0.01
RRI-total powers [ms^2^]	259.3 (14.4–1565.5)	29.5 (9.9–424.1)	1035.9 (151.9–1898.1)	* <0.01
**Sympathovagal balance**, median (IQR)				
RRI-LF/HF ratio	1.4 (0.8–2.0)	1.0 (0.5–1.4)	1.5 (1.2–2.4)	0.07

[*DCI*, delayed cerebral ischemia; *RRI*, R–R interval; *LF*, low frequency; *nu*, normalized unit; *SBP*, systolic blood pressure; *RMSSDs*, root mean square of successive differences; *HF*, high frequency; *SD*; standard deviation; *CV*, coefficient of variance]. * and bold indicate significant differences.

**Table 5:** Potential influence of the therapeutic regimen on day 5 on parameters of autonomic cardiovascular modulation.

	All Patients (*n* = 53)	DCI (*n* = 17)	No DCI (*n* = 36)	*p*-Values (*DCI vs. No DCI*)
**Nimodipine**, n (%)	53 (100)	17 (100)	36 (100)	1
**Vasopressor therapy**, n (%)	27 (50.9)	11 (64.7)	16 (44.4)	0.24
Norepinephrine	27 (50.9)	11 (64.7)	16 (44.4)	0.24
Other catecholamine	3 (5.7)	2 (11.8)	1 (2.8)	0.24
**Antihypertensivedrugs**, n (%)	11 (20.8)	1 (5.9)	10 (27.8)	0.08
Continuous infusion (syringe pump)	8 (15.1)	1 (5.9)	7 (19.4)	0.25
- Alpha-1 adrenoceptor antagonist	8 (15.1)	1 (5.9)	7 (19.4)	0.25
- Alpha-2 adrenoceptor agonist	1 (1.9)	0 (0.0)	1 (2.8)	1
- Dihydralazine	3 (5.7)	0 (0.0)	3 (8.3)	0.54
Oral drug therapy	6 (11.3)	1 (5.9)	5 (13.9)	0.65
- ACE inhibitor/ARB	6 (11.3)	1 (5.9)	5 (13.9)	0.65
- Betablocker	5 (9.4)	1 (5.9)	4 (11.1)	0.66
- Diuretic	2 (3.8)	0 (0.0)	2 (5.6)	0.56
- Calcium channel blocker	2 (3.8)	0 (0.0)	2 (5.6)	0.56
**Analgesic drugs**, n (%)	43 (81.1)	15 (88.2)	28 (77.8)	0.47
Opioids	43 (81.1)	15 (88.2)	28 (77.8)	0.47
Non-opioids	24 (45.3)	7 (41.2)	17 (47.2)	0.77
**Sedative drugs**, n (%)	34 (64.2)	13 (76.5)	21 (58.3)	0.23
Midazolam	30 (56.6)	11 (64.7)	19 (35.8)	0.55
Esketamine	21 (39.6)	8 (47.1)	13 (36.1)	0.55
Propofol	7 (13.2)	4 (23.5)	3 (8.3)	0.19
**Mechanical Ventilation**, n (%)	35 (66.0)	13 (76.5)	22 (61.1)	0.36

[*DCI*, delayed cerebral ischemia; *ACE*, angiotensin-converting enzyme; *ARB*, angiotensin II receptor blocker].

**Table 6:** Potential influence of therapeutic rescue strategies during ICU treatment on parameters of autonomic cardiovascular modulation.

	All Patients (*n* = 53)	DCI (*n* = 17)	No DCI (*n* = 36)	*p*-Values (*DCI vs. No DCI*)
**Cerebrospinal fluid drainage**, n (%)	40 (75.5)	15 (88.2)	25 (69.4)	0.18
External ventricular drain	32 (60.4)	12 (70.6)	20 (55.6)	0.46
Lumbar drain	30 (56.6)	12 (70.6)	18 (50.0)	0.27
**Intraventricular thrombolysis**, n (%)	22 (42.5)	9 (52.9)	13 (36.1)	0.39
**Intra-arterial vasospasmolysis**, n (%)	9 (17.0)	6 (35.3)	3 (8.3)	* 0.02
**Osmotic active therapy**, n (%)	8 (15.1)	7 (41.2)	1 (2.8)	* <0.01
Mannitol	5 (9.4)	4 (23.5)	1 (2.8)	* 0.03
Hypertonic saline	7 (13.2)	6 (35.3)	1 (2.8)	* <0.01
**TTM to apply normothermia**, n (%)	12 (22.6)	6 (35.3)	6 (16.7)	0.15
**Mechanical Ventilation**, n (%)	35 (66.0)	13 (76.5)	22 (61.1)	0.36

[*DCI*, delayed cerebral ischemia; *TTM*, target temperature monitoring]. * and bold indicate significant differences.

**Table 7:** Associations between biosignals as well as parameters of autonomic cardiovascular modulation on days 1 and 5 after spontaneous subarachnoid hemorrhage with functional outcome three to six months after hospital discharge.

	All Patients (*n* = 48)	Favorable Outcome (*mRS* ≤ 3; *n* = 33)	Unfavorable Outcome (*mRS* > 3; *n* = 15)	*p*-Values (*mRS* ≤ 3 *vs. mRS* > 3)
**DAY 5**				
**Biosignals**, median (IQR)				
Systolic blood pressure [mmHg]	139.0 (130.8–150.0)	139.3 (127.9–151.0)	136.6 (132.0–147.4)	0.80
Diastolic blood pressure [mmHg]	69.8 (60.3–76.1)	72.2 (65.9–77.2)	57.7 (50.3–73.5)	0.12
Heart rate [min^−1^]	883.4 (740.3–1032.2)	883.4 (732.3–1025.5)	888.6 (765.9–1126.9)	1
**Sympathetic modulation**, median (IQR)				
RRI-LF powers [ms^2^]	172.4 (8.0–1004.8)	286.1 (35.6–1034.9)	5.3 (0.8–375.8)	0.07
RRI-LF(nu) powers	0.6 (0.4–0.7)	0.6 (0.5–0.7)	0.4 (0.2–0.6)	* 0.03
SBP-LF powers [mmHg^2^]	5.4 (3.1–19.4)	8.1 (4.4–44.4)	3.7 (1.7–8.9)	0.12
**Parasympathetic modulation**, median (IQR)				
RRI-RMSSD [ms]	18.2 (5.8–33.7)	20.5 (13.2–38.0)	6.9 (4.5–30.5)	0.18
RRI-HF powers [ms^2^]	63.0 (6.9–559.3)	138.7 (2.1–570.3)	7.9 (5.5–294.5)	0.20
RRI-HF(nu) powers	0.4 (0.3–0.6)	0.4 (0.3–0.5)	0.6 (0.4–0.8)	* 0.03
**Total autonomic modulation**, median (IQR)				
RRI-SD [ms]	18.0 (4.7–44.2)	24.6 (11.8–46.0)	4.9 (3.0–23.5)	0.12
RRI-CV [%]	1.7 (0.6–4.7)	2.7 (1.5–5.3)	0.7 (0.4–1.9)	0.05
RRI-total powers [ms^2^]	254.5 (13.8–1593.5)	501.6 (71.6–1649.7)	13.0 (6.7–670.3)	0.08
**Sympathovagal balance**, median (IQR)				
RRI-LF/HF ratio	1.4 (0.7–2.0)	1.5 (1.1–2.7)	0.7 (0.2–1.4)	* 0.03

[*mRS*, modified ranking score; *RRI*, R–R interval; *LF*, low frequency; *nu*, normalized unit; *SBP*, systolic blood pressure; *RMSSDs*, root mean square of successive differences; *HF*, high frequency; *SD*; standard deviation; *CV*, coefficient of variance]. * and bold indicate significant differences.

## Data Availability

Data are available on request from the authors via mail to the corresponding authors, subject to approval and a data sharing agreement.

## Abbreviations

The following abbreviations are used in this manuscript:

SAH	subarachnoid hemorrhage
EBI	early brain injury
SBI	secondary brain injury
DCI	delayed cerebral ischemia
TCD	transcranial Doppler ultrasound
CT	computed tomography
HRV	heart rate variability
CAA	cerebral amyloid angiopathy
IMC	intermediate care unit
ICU	intensive care unit
mRS	modified Rankin scale
LTFU	lost to follow-up
IVH	intraventricular hemorrhage
TIA	transient ischemic attack
ACE	angiotensin-converting enzyme
ARB	angiotensin II receptor blockers
WFNS	World Federation of Neurosurgical Societies
NIHSS	National Institutes of Health stroke scale
mFS	modified Fisher scale
LOS	length of stay
RRI	R–R interval
BP	blood pressure
SBP	systolic blood pressure
DBP	diastolic blood pressure
SD	standard deviation
RMSSD	root mean square of successive difference
CV	coefficient of variation
TRS	trigonometric regressive spectral analysis
LF	low frequency
HF	high frequency
nu	normalized
Max.	maximum
TTM	target temperature monitoring
AUC	area under the curve
